# Optimal stride frequencies in running at different speeds

**DOI:** 10.1371/journal.pone.0184273

**Published:** 2017-10-23

**Authors:** Ben T. van Oeveren, Cornelis J. de Ruiter, Peter J. Beek, Jaap H. van Dieën

**Affiliations:** Department of Human Movement Sciences, Faculty of Behavioral and Movement Sciences, Vrije Universiteit Amsterdam, Amsterdam Movement Sciences, The Netherlands, Amsterdam, The Netherlands; University of Colorado Boulder, UNITED STATES

## Abstract

During running at a constant speed, the optimal stride frequency (SF) can be derived from the u-shaped relationship between SF and heart rate (HR). Changing SF towards the optimum of this relationship is beneficial for energy expenditure and may positively change biomechanics of running. In the current study, the effects of speed on the optimal SF and the nature of the u-shaped relation were empirically tested using Generalized Estimating Equations. To this end, HR was recorded from twelve healthy (4 males, 8 females) inexperienced runners, who completed runs at three speeds. The three speeds were 90%, 100% and 110% of self-selected speed. A self-selected SF (SF_self_) was determined for each of the speeds prior to the speed series. The speed series started with a free-chosen SF condition, followed by five imposed SF conditions (SF_self_, 70, 80, 90, 100 strides·min^-1^) assigned in random order. The conditions lasted 3 minutes with 2.5 minutes of walking in between. SF_self_ increased significantly (p<0.05) with speed with averages of 77, 79, 80 strides·min^-1^ at 2.4, 2.6, 2.9 m·s^-1^, respectively). As expected, the relation between SF and HR could be described by a parabolic curve for all speeds. Speed did not significantly affect the curvature, nor did it affect optimal SF. We conclude that over the speed range tested, inexperienced runners may not need to adapt their SF to running speed. However, since SF_self_ were lower than the SF_opt_ of 83 strides·min^-1^, the runners could reduce HR by increasing their SF_self_.

## Introduction

Running speed is the product of stride frequency (SF) and stride length (SL), and both are shown to increase when runners increase their speed. SF, expressed in strides per minute (strides·min^-1^), describes the duration of a complete stride cycle (left and right step). SF consequently relates to many biomechanical aspects of running [1–15]. Hence, SF has received considerable attention from both scientific and practical perspective. Although many sports-watches provide instantaneous SFs, they do not yet provide feedback about the SF at which energy cost for the individual runner is minimized: SF_opt_, the optimal SF at a given speed. In practice, as a rule of thumb and independent of running speed, runners are often advised to run at 90 strides·min^-1^ [16]. This recommendation is based on the observation that, for all running distances, elite runners seem to use a SF of at least 90 strides·min^-1^. However, it is doubtful that this SF is optimal for every runner at every speed, as 90 strides·min^-1^ is substantially higher than most self-selected stride frequencies (SF_self_) and even higher than SF_opt_ reported in literature [4,17]. Cavanagh et al [18] suggested that only (individual) physiological evidence of a discrepancy between preferred and SF_opt_ should be used to advise changes in SF.

To calculate SF_opt_, previous studies typically imposed SF or SL and recorded oxygen consumption while participants ran at a constant submaximal speed [4,19–23]. The relation between SF and oxygen consumption was fitted per individual using a second order polynomial, resulting in a u-shaped relation, where the SF with minimal oxygen consumption was considered to be optimal [1,19,21–23]. These studies showed that experienced runners tend to run with SFs close to their SF_opt_ [19,23,24], while inexperienced runners appear to run with SFs below their SF_opt_ [19]. As an alternative to measuring oxygen consumption, De Ruiter et al [19] showed that heart rate (HR) can also be used to determine the SF_opt_.

Several biomechanical mechanisms may explain the reduction of energy cost that occurred when individual runners slightly increased their SFs. For example, higher SFs are associated with reduced vertical oscillations [1–3], shorter ground contact [1,2], increased leg stiffness [1] and reduced horizontal braking forces [4–6], which are all factors suggested to be important for economical running [5,8]. In addition, increasing SF changes the kinetics in the gait cycle change in ways that have been suggested to reduce injury risk [6,13–15]. For example, higher SFs reduce anterior foot placement [3,4], impact forces [9], vertical accelerations [4,7,11] [10], knee extension moments at initial contact [12] and negative energy at the hip and knee [3,7]. Among runners injury incidence rates as high as 79.3% have been reported [25] and changing running technique may be effective to reduce the injury risk. Inexperienced runners may be particularly susceptible to injuries because of their lower tolerance to impact forces [26,27].

**Fig 1:**
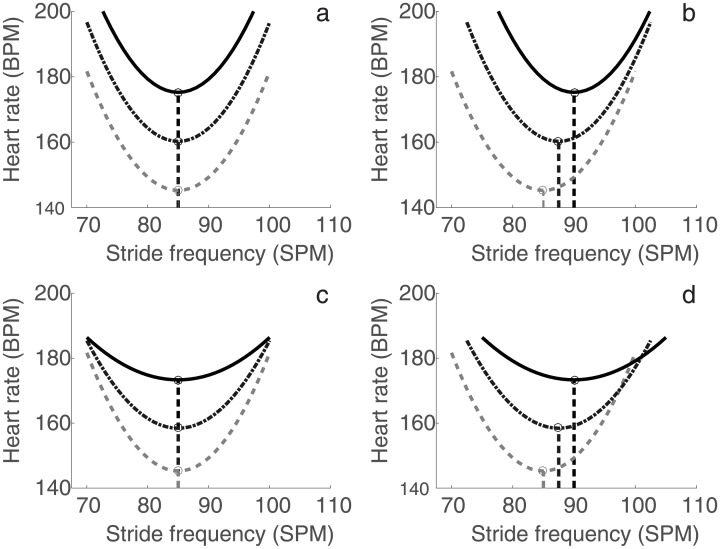
Hypothetical models to evaluate the effect of speed on the relation between stride frequency and heart rate. The dashed line (V90%), dash-dot (V100%) and solid line (V110%) respectively represent three increasing speed conditions.

HR, speed and SF are measured by many commercially available sports devices, which would in principle allow runners to determine their own SF_opt_. Reviewing results from previous studies revealed that both SF_self_ and SL increase when individual runners increase their speed [1,4,9,14,19,21,22,28–33]. Therefore, it can be expected that SF_opt_ also increases with speed (Fig 1c and 1d). To attain higher speeds, energy cost per unit of time will increase. HR consequently approaches its physiological maximum and the parabolic relation between SF and HR may flatten (Fig 1a and 1b). A relatively steep parabola with a profound optimum may then be apparent at low speeds. At higher speeds, the curve is expected to flatten, which would make the estimation of SF_opt_ less robust. However, we hypothesize that for normal training intensities this flattening is of no significance, since for healthy individuals, heart rate increases linearly with exercise load and only plateaus just before maximal oxygen consumption is reached [34]. The effect of speed on SF_opt_ may seem obvious. However, to our knowledge, the effect of speed on the calculation of SF_opt_ has never been tested systematically. Understanding the effect of speed on the SF-HR relation is required to develop a method to provide runners with feedback on their SF_opt_ during training.

The aim of this study is to understand the practical consequence of changing speed on the relation between SF and HR in running. To this end, we use a group-based analysis. We hypothesize that within a speed range that is representative and attainable for inexperienced runners: 1) a speed-specific parabolic relation exists between SF and HR; 2) SF_opt_ increases with speed; 3) the parabolic SF–HR relation does not flatten at higher speeds; 4) inexperienced runners select SFs below the determined optimum.

## Methods

Four models were tested to evaluate the effect of speed on the calculation of SF_opt_ (see Fig 1). SF_opt_ was defined as the SF where HR (response variable) was minimal according to the model. The models are described with the following equations:

$$HR\;=\;b_{0}+\;b_{1}∙SF+\;b_{2}∙{SF}^{2}+b_{3}∙V$$ (Model 1)

$$HR\;=\;b_{0}+\;b_{1}∙SF+\;b_{2}∙{SF}^{2}+b_{3}∙V\;+b_{4}∙SF∙V$$ (Model 2)

$$HR\;=\;b_{0}+\;b_{1}∙SF+\;b_{2}∙{SF}^{2}+b_{3}∙V\;+b_{5}∙{SF}^{2}∙V$$ (Model 3)

$$HR\;=\;b_{0}+\;b_{1}∙SF+\;b_{2}∙{SF}^{2}+b_{3}∙V\;+b_{4}∙SF∙V+\;b_{5}∙{SF}^{2}∙V$$ (Model 4)

Model 1 assumes a parabolic relationship (b_1_, b_2_) with a speed-dependent offset (b_3_). Model 2 assumes that SF and consequently SF_opt_ increases linearly with speed (b_4_). Model 3 assumes that speed changes the parabolic relation between SF and HR (b_5_). More specifically, it reflects a flattening of the curve at higher speeds. Model 4 assumes both a linear speed dependency of SF as described by Model 2 (b_4_) and a flattening of the curve as described by Model 3 (b_5_). The model regression coefficients were tested for significance in order to establish their contribution to the fit. Models with significance on all predictors are potentially valid.

### Participants

Twelve healthy (4 males, 8 females) inexperienced runners participated (23.3 ± 3.4yrs, 175 ± 1.1cm, 69.6 ± 13.0kg) in the present study, after given written informed consent approved by the Institutional Review Board at the Vrije Universiteit Amsterdam. The study was approved by the local ethics committee (in Dutch: Ethische Commissie Bewegingswetenschappen) in accordance with the guidelines set out in the Helsinki Declaration regarding human research.

Participants ran no more than once a week and participated at least once a week in sports activities that did not involve running (4.8 ± 2.6 training hours per week). Inexperienced runners were chosen for this study since they were expected to benefit most from altering their SF. [19]

### Instrumentation

Participants ran on a treadmill (N-Mill, ForceLink, Culemborg, Nederland). Strides were detected using a custom written Matlab program analysing the tri-axial acceleration data from sensors (MPU-9150, +/- 16.0g, 500Hz, 35 x 25 x 11 mm, Invensense, San Jose, USA) placed on the heels of the participants. HR was measured using a HR monitor (Suunto t6d, Vantaa, Finland). To synchronize the SF-data with the HR-data, participants jumped causing a peak in the acceleration signal, while simultaneously starting the HR-monitor.

### Protocol

All measurements took place on the same day. Prior to testing, self-selected running speed (V_100%_) was determined as follows. The participants ran for 5 minutes at a speed of 2.22 m·s^-1^ (8 km·h^-1^). Subsequently, every 20 s speed was increased by 0.14 m·s^-1^ (0.5 km·h^-1^). Participants were asked to indicate when they reached a speed that they felt able to sustain for 10 minutes with moderate to strong effort ratings on the RPE-scale. The protocol consisted of three separate speed series in fixed order: (i) Starting with the self-selected speed (V_100%_), followed by (ii) 90% of self-selected speed (V_90%_) and finally, (iii) 110% of self-selected speed (V_110%_). This was done to minimize the risk of participants not being able to complete the protocol due to the expected fatigue expected at V_110%_.

Within each speed series, the participants ran first for 3 minutes without instructions to get familiarized with running on the treadmill and to determine the SF_self_. Where SF_self_ was calculated as the mean SF over the last minute of familiarization trail. After the 3 minutes familiarization, participants had 5 minutes of rest followed by six blocks of 3 minutes. The first block was intended as warming-up without an imposed SF. The consecutive blocks were conditions with imposed SFs (i.e. 70, 80, 90, 100 strides·min^-1^ and SF_self_) administered in randomised order (without replacement) for each participant. Thus, the order of SF conditions differed among participants, but the SF condition order at each of the three speeds was kept consistent for each participant.

Each block was followed by 2.5 minutes of walking at 1.11 m·s^-1^ (4 km·h^-1^). Participants rested for 15 minutes after each of the speed series. SF was imposed by lines projected on the treadmill perpendicular to the running direction (visually similar to a zebra crossing) using a projector. The lines were projected across the full width of the treadmill and from 2 m in front of the participants to 1 m behind the centre of the treadmill and approached the runner with the speed of the treadmill. Participants could choose to either step on or step between the projected lines as long as they were consistent.

### Data analysis

Data were synchronized and pre-processed using Matlab 2015. For every 3-minute block, the median SF and median HR were calculated over the last minute. Generalized Estimating Equation (GEE) modelling was used to evaluate the four models. The dependency of the variables speed, SF, and HR is expected to differ between individuals [23]. By choosing an exchangeable correlation structure, GEE offers the possibility to assume that all observations within the same individual are equally correlated. GEE is a group-based approach that can take the dependency of observations within individuals over conditions into account. The GEE does not assume normally distributed data. The GEE was performed using SPSS 22.0. In order to measure how well the participants were able to run at the imposed stride frequencies, a percentage error (SF_error_) was calculated from the imposed versus the observed SF (Eq 1).

$${SF}_{error}\;=\;({SF}_{observed}-\;{SF}_{imposed})/\;{SF}_{imposed}$$ (1)

Differences in SF_self_ between speeds were tested using a Friedmans’s test with a significance level of 0.05. Post hoc analysis was done using Wilcoxon signed ranks test. The protocol was designed to reduce exercise load while simultaneously minimize the variation in HR between blocks when the SF-conditions were not present. Nevertheless, exercise load was relatively high and body temperature was expected to increase. Consequently, we did not expect that HR would completely level off during the last minutes of the exercise steps. To indicate the level of steady-state, the slope of HR during the last minutes of the exercise steps was calculated using a bi-square linear fit.

## Results

Only in Model 1 were all parameters found to be significant (Table 1) and thus the relation was best described by Model 1. Model 1 indicated that the relation between SF and HR can be described by separate parabolic relations at all speeds. Model 2 predicted that SF_opt_ would increase with speed, but the predictor did not appear to be significant (Model 2, b_4_: p = 0.090). Model 3 predicted a flattening of the curve at higher speeds, but also the quadric SF-term did not significantly interact with speed (Model 3, b_5_: p = 0.080). Finally, Model 4, predicted both an increase of the optimum and a flattening of the curve with increasing speed, but also this model did not significantly improve the fit (Model 4, b_3_: p = 0. 077, b_4_: p = 0.056, b_4_: p = 0.053).

**Table 1:** Significance of the model parameters.

	Model 1			Model 2			Model 3			Model 4		
	B	Std.Error	P-value	B	Std.Error	P-value	B	Std.Error	P-value	B	Std.Error	P-value
b_0_	370.595	51.845	p<0.01	272.616	53.810	p<0.01	324.864	42.839	p<0.01	1436.062	591.728	0.015
b_1_ (SF)	-6.223	1.181	p<0.01	-5.171	1.026	p<0.01	-6.511	1.242	p<0.01	-33.138	14.716	0.024
b_2_ (SF^2^)	0.038	0.008	p<0.01	0.039	0.008	p<0.01	0.048	0.012	p<0.01	0.206	0.091	0.024
b_3_ (V)	22.016	3.833	p<0.01	63.669	2.144	p<0.01	44.022	10.413	p<0.01	-379.983	214.795	0.077
b_4_ (SF * V)			-	-0.504	0.298	0.090	-		-	10.155	5.315	0.056
b_5_ (SF^2^ * V)	-		-	-		-	-0.003	0.002	0.081	-0.063	0.033	0.053

The parameter terms *b*_*0-5*_ are based on the following equation:

$HR\;=\;b_{0}+\;b_{1}∙SF+\;b_{2}∙{SF}^{2}+b_{3}∙V\;+b_{4}∙SF∙V+\;b_{5}∙{SF}^{2}∙V$

Based on Model 1, the heart rate at SF_opt_ can be expressed as: HR = 370.59 + -6.223·SF + 0.0375·SF^2^ + 22.016·V. The SF_opt_ is obtained as the minimum in the HR-SF relationship, calculated as the SF derivative of HR. Therefore, SF_opt_ = 6.223 / (2*0.0375), which yields SF_opt_ = 83 strides·min^-1^ for all speeds used in this study. Due to technical problems, data of one participant were missing at V_100%_ and V_110%_, while for another participant the data at V_90%_ were missing, resulting in eleven subjects per speed condition and 69, 64, and 61 observations, respectively.

**Fig 2:**
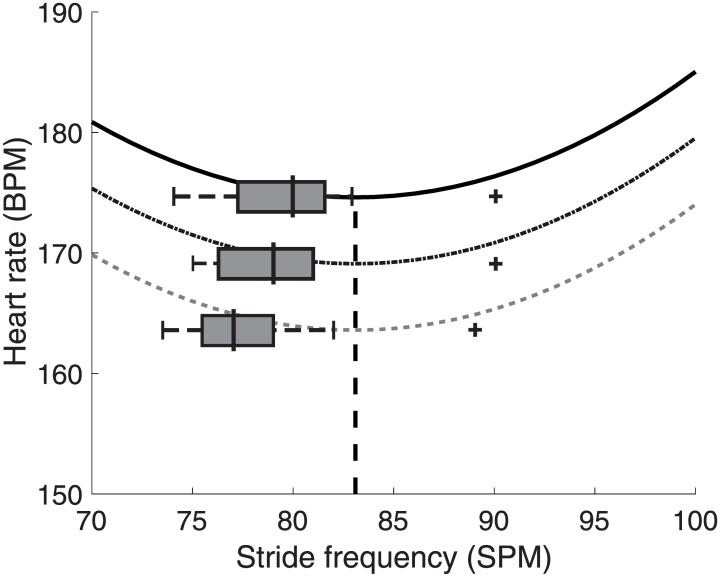
The relation between SF and HR for each speed series based on Model 1. From bottom to top V_90%_, V_100%_, V_110%_. The central line in the boxplot represents the median, the edges of the box are the 25th and 75th percentiles, and the whiskers extend to ±1.5 of the interquartile range. The outliers (+) beyond this range belong to a single participant. An optimum of 83 strides·min^-1^ was calculated using the parameters of Model 1.

Median speed values (25 and 75 percentiles) for the three speed categories respectively were: 2.38 (2.38–2.56), 2.6 (2.5–2.9), and 2.9 (2.8–3.1) m·s^-1^, with SF_self_: 77 (75.5–78.5); 79 (76.5–81.0); 80 (77.5–81.0) strides·min^-1^ and HR: 166 (155–178); 172 (165–176.); 177(167–180) beats·min^-1^. SF_self_ differed significantly between speed conditions (X^2^(2) = 6.686, p = 0.035) and post-hoc analysis showed a significant difference only between V_90%_ and V_100%_ (Z = -2.354, p = 0.019). Note that at each speed, SF_self_ was below the optimum of 83 strides•min^-1^ predicted by Model 1 (Fig 2) by 6%, 5%, 2% for V90%, V100%, and V110%, respectively. Only one participant ran at about 90 strides·min^-1^ at all speeds, which clearly was above the calculated optimum of 83 strides·min^-1^

**Fig 3:**
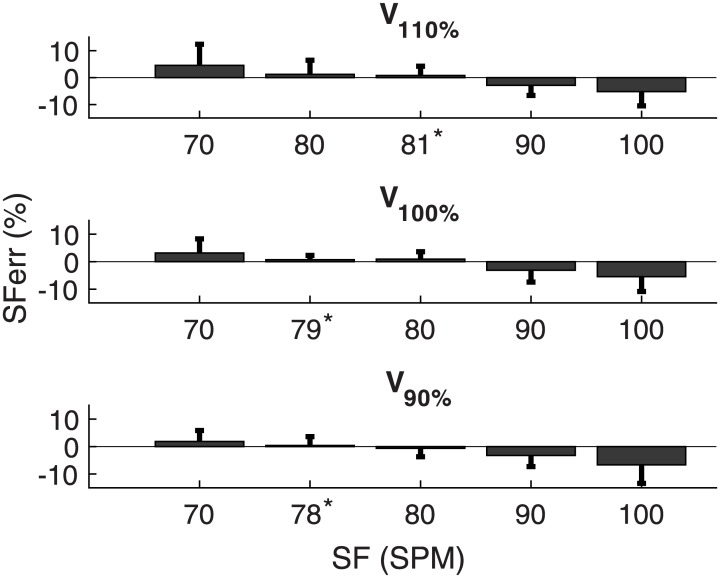
Average error scores calculated from the observed stride frequency (SF) relative to the imposed SF. The asterisk denotes the average self-selected SF.

Participants did not always succeed in running at the imposed SFs (Fig 3). Deviations between the calculated SF and the imposed SF were similar between conditions 2.5%±4% (mean±sd), with a tendency towards higher errors in the V_90%_ and V_110%_ conditions compared to the V_100%_-condition. Moreover, errors increased for more extreme SF-conditions (e.g., 70 and 100 strides·min^-1^).

## Discussion

The aim of this study was to examine the effect of speed on the relationship between SF and HR within an ecologically valid speed range for inexperienced runners. For all speeds, the SF-HR relation could be described by a parabolic curve; these curves shared an SF_opt_ at 83 strides·min^-1^. The curve did not flatten as expected and SF_opt_ did not significantly increase with speed. Therefore, it was not needed to extend Model 1 with additional parameters as in Models 2–4. To our knowledge, the effect of speed on the calculation of SF_opt_ has not been studied before. Previous studies have used direct curve-fitting procedures on individual observations [19,20,23,35]. Instead, in this study we used a group-based analysis (GEE) as a more robust alternative.

The optimum of 83 strides·min^-1^ determined here, does not differ much from previously calculated SF_opt_. In a group of inexperienced runners, De Ruiter et al [19] found an optimum of 84.9 strides·min^-1^ at an average speed (2.67m·s^-1^) comparable to the average self-selected speed in the present study. The difference of 1.9 strides·min^-1^ is relatively small and can be attributed to differences in the analytical approach, methodology, and variance between subjects [19,23]. Note that in both studies, SF_opt_ was considerably lower than the 90 strides·min^-1^ proposed by Daniels [16], which is currently often used as a reference in practice. As a rule of thumb, it seems more appropriate to advise inexperienced runners with speeds between 2.4 to 2.9 m·s^-1^ to run at SF near 83 strides·min^-1^.

The speed range used in this study was intended to reflect common exercise intensities and based on a self-rated intensity score during a pre-test. HR was used as an estimate for intensity and energy cost. HR ranged on average between 166 and 176 beats·min^-1^ over the speeds, which seems an appropriate reflection of regular endurance training intensities. HR did not completely level off during the SF-conditions. However, the calculated HR slope over the last minute was small (1.4 beats.min^-1^). Besides, even during truly steady state conditions, thus while running below lactate threshold and with longer (5 min) exercise blocks, HR does not completely level off [36]. Therefore, we believe that HR values and thus the speed range used in this study sufficiently reflect training intensities of this group of runners.

Still, the speed range tested was relatively small (2.4 to 2.9m·s^-1^, i.e. 8.6 to 10.4 km·h^-1^). Measured SF_self_ changed significantly with speed, but only between the first two speeds (V_90%_ and V_100%_). The calculated SF_opt_ did not change with speed, but the interaction effect of speed and SF was close to significance (p = 0.09). In accordance with the non-significant interaction between SF and speed in the present study, Weyand et al [33] also did not observe significant changes in SF_self_ within a speed range of 2 to 4m·s^-1^. Only for larger speed ranges did SF_self_ increase significantly [33]. Mercer et al [22] also did not find a significant effect of speeds from 3.13, 3.58, 4.02 m·s^-1^ on the relation between SF and oxygen consumption. Note that their goal was not to determine an SF_opt_, but still they used three (non-randomized SF) conditions. In the present study, the median values of SF_self_ at the different speeds revealed a trend and the p-values for the interaction terms of speed on SF in Models 2 and 4 were small. Post-hoc analysis revealed that the study was slightly underpowered and hence a type II error cannot be excluded. Therefore, Model 1 may not hold when tested over a larger speed range or with a larger group of participants. Especially, for more experienced runners the speed range will likely be larger and it is not unlikely that speed will affect their SF_opt_.

Similarly, although we did not find flattening of the SF-HR relation at higher speeds in the present study, a flattening may occur in more experienced runners who are able to run at higher percentages of maximal HR for longer durations.

Interestingly, our results suggest that the participants could immediately reduce their heart rates when they would be running at higher SFs, closer to 83 strides·min^-1^. Similarly, in previous studies [4,19–21], runners would reduce oxygen cost immediately by increasing their SF. It remains to be investigated whether the energy sparing effect will increase even more after a period of habituation and/or training at higher SFs. As far as we know, for endurance intensities, there are no studies suggesting that runners should run at SFs other than determined by the energetic optimum. Since, it is currently unknown what the reason is for inexperienced runners to run at SFs below the energetic optimum, understanding of what limits adaptation of SF towards the optimum may help to improve learning strategies. It might be argued that coordination may limit the attainable SF, as higher SFs require faster muscle recruitment and derecruitment. From this study and also from the pilot experiments (where a metronome was used) it became clear that some runners did find it very hard to stabilize newly imposed SFs. Participants also made more errors at the extreme imposed SFs. Similar errors were found in a previous study in which SF was imposed by the use of a metronome [18]. However, the low number of errors in the 80 strides•min^-1^ condition suggests that running at SFs of 83 strides•min^-1^ is unlikely to be hampered by limitations in coordination.

There are many individual factors that could explain the large inter-individual differences in SF_self_. For example, SF at a given speed has been found to decrease with fatigue [37] and in a subject-specific manner [20]. In addition, damping properties of footwear [1,38–40] and slope [31,41] could affect SF. Effects of anthropometric factors such as body composition or leg length are small in comparison with other factors [28]. In line with this, it was recently shown that a change in SF_opt_ resulted only after adding as much as 1 kg mass to each ankles [42]. The different factors can be categorized in individual characteristics (e.g., neuromuscular control, fibre type, body weight or mass), time-varying variables (e.g., speed, fatigue), and environmental circumstances (e.g., (shoe) damping, surface, slope, hypoxia, heat [43]). Note that most of these factors will be reflected in individual energy cost and that only some of them will vary sufficiently within a training to take into account when providing feedback. Nevertheless, it may be worthwhile to investigate their effects on the SF-HR relation while taking speed into consideration to understand differences between groups. Given the high inter-individual differences in SF_self_, the many possible factors influencing SF, not to mention the importance of variability [44], the advice to run at 83 strides·min^-1^ should not be generalized to all individuals or to all running conditions.

In the current study, we used a group-based approach in order to get a robust fit. Future studies could apply the proposed models to a larger population, which would allow for better generalization, or study the effects speed over a larger speed range to understand the differences in running experience. In addition, it is worthwhile to study the reason for inexperienced runners to select SFs below the energetic optimum, to what extent, and how quickly these runners adapt SF_self_ towards the energetic optimum with specific training. Nevertheless, since commercially available running equipment can already measure speed (by GPS), HR, and SF, it may already be possible to use the logged data from such equipment to determine SF_opt_.

## Conclusion

We conclude that SF_opt_ is relatively stable at the speeds used by inexperienced runners. The speed range in this study was determined individually and was intended to reflect the habitual endurance running intensities of inexperienced runners. For almost all the participants, the SF_self_ was substantially lower than the optimum of 83 strides·min^-1^. Therefore, the results suggest that inexperienced runners can obtain direct energetic benefit from increasing their SFs. It seems of lesser importance that runners adapt their SF instantaneously to specific speeds, since the commonly used speed range of inexperienced runners will be rather limited. Intra-individual differences are high and contextual differences may constrain SF_opt_, therefore, the generalized advice to run at 83 strides·min^-1^ should be avoided and feedback on SF should be determined individually. In addition, the current study does not rule out the possibility that SF_opt_ may increase with speed over larger speed ranges. Future studies could extend this work by applying the models on data logged by commercial devices to provide runners with individualized feedback on their SF_opt_.

## Supporting information

S1 File SFxHRxV. Data used for the model fitting. With respectively subject number, condition order, speed (m/s), stride frequency (spm), heart rate (bpm).
(XLSX)
